# Clinical Validity of Imatinib Therapeutic Drug Monitoring in a Real-World Italian Cohort of Gastrointestinal Stromal Tumors and the Role of Patients’ Sex

**DOI:** 10.3390/pharmaceutics18080968

**Published:** 2026-08-07

**Authors:** Sara Gagno, Angela Buonadonna, Eleonora Cecchin, Arianna Fumagalli, Bianca Posocco, Giovanni Canil, Riccardo Cecchin, Michela Guardascione, Marcella Montico, Fabio Puglisi, Erika Cecchin

**Affiliations:** 1 Experimental and Clinical Pharmacology, Centro di Riferimento Oncologico di Aviano (CRO), IRCCS, 33081 Aviano, Italy; sgagno@cro.it (S.G.); eleonora.cecchin@cro.it (E.C.); giovanni.canil@cro.it (G.C.); riccardo.cecchin@cro.it (R.C.); ececchin@cro.it (E.C.); 2 Department of Medical Oncology, Centro di Riferimento Oncologico di Aviano (CRO), IRCCS, 33081 Aviano, Italy; abuonadonna@cro.it (A.B.); arianna.fumagalli@cro.it (A.F.); michela.guardascione@cro.it (M.G.); fabio.puglisi@cro.it (F.P.); 3 Unit of Cancer Epidemiology, Centro di Riferimento Oncologico di Aviano (CRO), IRCCS, 33081 Aviano, Italy; marcella.montico@cro.it; 4 Department of Medicine, University of Udine, 33100 Udine, Italy

**Keywords:** imatinib, GIST, TDM, exposure, toxicity, sex

## Abstract

**Background**: Substantial inter-individual variability in imatinib systemic exposure may influence both efficacy and toxicity. Based on recommendations of the International Association for Therapeutic Drug Monitoring and Clinical Toxicology (IATDMCT), the IRCCS-CRO of Aviano started in 2018 offering physicians the opportunity to monitor plasma concentrations of imatinib and its active metabolite, norimatinib, as part of a diagnostic service for patients with Gastrointestinal Stromal Tumor (GIST). This study aims to evaluate the potential clinical utility of imatinib Therapeutic Drug Monitoring (TDM) in predicting response and toxicity in a real-world cohort of 63 patients with GIST. **Methods**: Imatinib and its active metabolite norimatinib trough concentrations (C_min_) were quantified using a validated LC–MS/MS method. Associations between drug exposure and clinical–demographic characteristics, treatment-related toxicity, and progression-free survival (PFS) were evaluated. **Results**: A total of 437 plasma samples from 63 patients were collected. Marked inter- and intra-patient variability in imatinib exposure was observed (43% and 31% respectively); 67% of patients receiving 400 mg/day had a C_min_ below the recommended target concentration (1100 ng/mL). Female sex was significantly associated with higher imatinib exposure (median C_min_ 1114 vs. 786 ng/mL in males; *p* = 0.0018). Combined age–sex analysis showed a significant difference between women and men < 60 years (1041 vs. 668 ng/mL; *p* = 0.0068, Bonferroni-adjusted). A strong exposure–toxicity relationship emerged, with higher imatinib levels in samples collected at toxicity occurrence vs. the others (1728 vs. 927 ng/mL; *p* < 0.0001), without a direct association between sex and toxicity. No significant association between C_min_ and PFS was observed; however, disease progression was more frequent in patients with C_min_ < 500 ng/mL and absent in those with C_min_ >1500 ng/mL (60% vs. 0%, respectively). **Conclusions**: TDM was found to be a potentially useful tool for predicting clinically relevant toxicity and outcome. Sex and age significantly influence exposure, supporting tailored dosing strategies.

## 1. Introduction

Gastrointestinal stromal tumors (GISTs) are rare mesenchymal tumors that arise mainly in the stomach and small intestine and are commonly driven by activating mutations in c-KIT or PDGFRA, which determine sensitivity to targeted therapies [1]. The tyrosine kinase inhibitor imatinib has markedly improved outcomes, achieving response rates up to 80% [2] and median survival over 5 years [3]. Imatinib is currently approved in the neo-adjuvant, adjuvant, and advanced settings of GIST at the standard dose of 400 mg/day, with dose escalation to 800 mg/day recommended for patients with *c-KIT* exon 9 mutations in the advanced setting.

Despite the remarkable improvements in patients’ survival gained with imatinib, the clinical management of patients with GIST remains challenged by marked inter-individual variability in treatment response and tolerability, with treatment failure—due to either disease progression or treatment-limiting toxicity—still occurring. While disease progression is mainly driven by secondary resistance mutations in *c-KIT* or *PDGFRA*, inadequate systemic drug exposure represents a potentially modifiable and often overlooked contributor to suboptimal outcomes. Conversely, although imatinib is generally well tolerated, clinically relevant adverse events may lead to dose reduction or permanent discontinuation in some patients.

Pharmacokinetic studies have consistently demonstrated a wide inter-patient variability in imatinib systemic exposure, with coefficient of variation of 47–75% [4]. This variability is largely explained by differences in absorption, cytochrome P450-mediated metabolism, drug–drug interactions (DDIs), treatment adherence, and other patient-related factors, such as their pharmacogenetic background [5,6,7]. In addition, sex-related differences have been increasingly recognized as a potential source of pharmacokinetic variability. In general, females exhibit lower body weight, higher body fat percentage, and differences in hepatic enzyme activity and transporter expression, including CYP3A4, which may result in higher dose-normalized exposure for several drugs [8]. With regard to imatinib, in population pharmacokinetic analyses, sex hassa been associated with variability in apparent oral clearance [9,10], and observational data indicate that women may exhibit higher dose-normalized plasma concentrations of imatinib compared with men, partly attributable to differences in body weight [4,11].

Clear exposure–response and exposure–toxicity relationships have been described for imatinib. In a pivotal study by Demetri et al., patients with steady-state trough concentrations (C_min_) below 1100 ng/mL experienced a significantly shorter median time to progression compared with those achieving higher concentrations (11.3 vs. >30 months, *p* = 0.0029) [12]. This threshold is consistent with the exposure of imatinib associated with clinical response in chronic myeloid leukemia (1000 ng/mL) [13,14,15]. Subsequent real-world studies in GIST confirmed an exposure–efficacy relationship, although a lower C_min_ threshold (760 ng/mL) was associated with the most significant progression-free survival (PFS) benefit, with only a non-significant trend observed at 1100 ng/mL [16].

In parallel, an exposure–toxicity relationship has also been described for imatinib. Higher plasma concentrations have been associated with an increased incidence of adverse events, including fluid retention, gastrointestinal toxicity, myelosuppression, fatigue, and dermatological reactions, both in GIST and CML populations [13,14,17,18,19]. Together, these findings suggest the existence of a relatively narrow therapeutic window for imatinib and provide the rationale for individualized exposure assessment.

Therapeutic drug monitoring (TDM), i.e., the clinical practice of measuring and interpreting the drug levels in patients’ bloodstream aimed at optimizing drug exposure, has therefore emerged as a potential tool to manage the marked inter-individual pharmacokinetic variability of imatinib and to support personalized treatment. A prospective nationwide implementation study by IJzerman et al. demonstrated the feasibility of routine imatinib TDM in the Netherlands, showing that TDM increased the proportion of patients achieving adequate exposure (≥1100 ng/mL) and could be successfully integrated into daily clinical practice across multiple centers [4].

In line with these findings, the International Association of Therapeutic Drug Monitoring and Clinical Toxicology (IATDMCT) published a consensus guideline in 2021 clearly recommending imatinib TDM and defining target C_min_ values (≥1100 ng/mL in GIST and >1000 ng/mL in CML) [20].

Based on IATDMCT recommendations, the National Cancer Institute—CRO Aviano offers TDM diagnostic service for selected oral anticancer agents, including imatinib, to support oncologists in their routine clinical practice, including the management of complex scenarios such as suboptimal response, suspected DDIs, or adherence issues.

The aim of the present study is to evaluate the clinical utility of imatinib TDM in predicting response and toxicity in a mono-centric real-world cohort of 63 Italian patients with GIST treated across different clinical settings. Specifically, we evaluated the association between imatinib exposure and clinical–demographic variables, treatment-related toxicity, and PFS, with the goal of generating real-world evidence to support the potential role of TDM in the clinical management of GIST patients.

## 2. Materials and Methods

### 2.1. Patients’ Enrollment

Since 2018, the National Cancer Institute of Aviano has offered physicians the opportunity to monitor plasma concentrations of imatinib and its active metabolite, norimatinib, as part of a diagnostic service for patients with GIST. This service was intended as a supportive tool for therapy management; however, no dose adjustments were recommended based on the measured imatinib concentrations. Patients accessing this service were asked to provide written informed consent to prospectively participate in at least one of the following observational clinical studies: (1) Pilot study to evaluate the feasibility of an innovative approach to monitor patients with gastrointestinal stromal tumor treated with imatinib (EuDRACT number: 2017-002437-36; protocol ID: CRO-2017-19; approval date: 21 December 2017; CEUR opinion: CEUR-2017-Sper-125-CRO); and (2) CRO–Aviano integrated pharmacological counseling program (ClinicalTrials.gov identifier: NCT06822959; protocol ID: CRO-2022-14; approval date: 12 April 2022; CEUR opinion: CEUR-2022-Os-65).

Both studies were approved by the local Ethics Committee (Comitato Etico Unico Regionale del Friuli Venezia Giulia, CEUR) and conducted in accordance with the principles of the Declaration of Helsinki (latest revision).

Adult patients (≥18 years) with a diagnosis of GIST treated with imatinib (either starting or already on treatment) at any daily dose (100–800 mg) and in any treatment setting (neo-adjuvant, adjuvant, first-line, second-line, or re-challenge) who accessed the diagnostic service and were enrolled in the above-mentioned studies were considered eligible for the present analysis.

### 2.2. Blood Sampling Collection and Measurement of Imatinib and Norimatinib Concentrations

Blood samples were collected during routine follow-up visits. Samplings were generally performed monthly during the first three months of treatment and every three months thereafter or at each routine clinical visit when visits occurred at intervals longer than three months. All samples were collected at steady-state (at least after four consecutive days of continuous treatment at a stable dose), and preferentially at 24 h from the last imatinib intake in order to capture the trough concentration (C_min_). Blood samples were collected into 4.9 mL K-EDTA tubes and centrifuged at 2500× *g* for 10 min at 4 °C to obtain plasma for TDM analysis. Plasma samples were stored at −80 °C until analysis.

At the time of sampling, the following information was systematically recorded: body weight and height, imatinib daily dose, date and time of last drug intake, date and time of blood draw, concomitant medications or herbal supplements, fasting status, recent infections, smoking habits, and alcohol consumption. For samples not collected at the predefined time window, imatinib C_min_ was estimated using a previously validated algorithm [21]:

$$Cmin=Cmeasured\times \;0.5^{\frac{dosing\;interval-Time\;After\;Dosing}{t1/2}}$$

where t1/2 is the drug half-life (18 h for imatinib and 40 h for norimatinib).

Imatinib and norimatinib plasma concentrations were quantified using a liquid chromatography–tandem mass spectrometry (LC–MS/MS) method validated in accordance with the most recent European Medicines Agency (EMA) [22] and U.S. Food and Drug Administration (FDA) [23] bioanalytical guidelines, and accredited by ACCREDIA for diagnostic use according to ISO 15189 [24] quality standards. Patient samples were analyzed in duplicate, and the mean concentration value was used for association analyses.

### 2.3. Study Design

**Figure 1:**
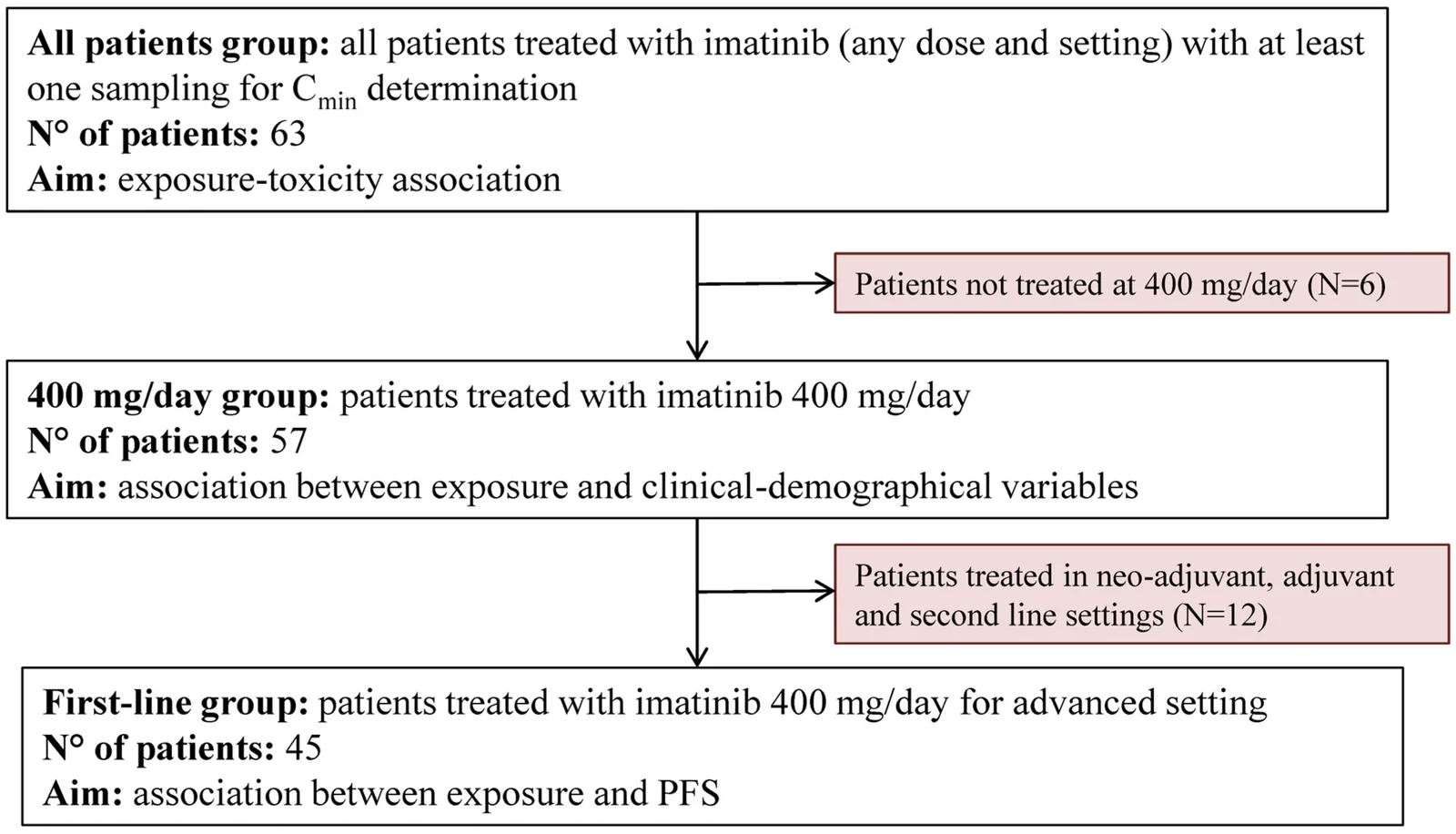
Flow-chart of the study design.

A schematic representation of the study design is provided in Figure 1.

In the overall study cohort (“All patients” group, N = 63 patients), the association between imatinib (and norimatinib) exposure and clinically relevant toxicity was explored by assessing the difference in exposure between “toxic” and “non-toxic samples”. “Toxic samples” were defined as samples collected within a ±6-month window around the occurrence of a clinically relevant toxicity while samples obtained outside this time window or from patients without clinically relevant toxicity were considered “non-toxic samples”. Clinically relevant toxicities were defined as any imatinib-related adverse event requiring dose reduction, temporary treatment interruption, or permanent discontinuation.

Analyses exploring the relationship between imatinib (and norimatinib) exposure and clinical–demographic variables were conducted in the subgroup of patients receiving a homogeneous daily dose of 400 mg (“400 mg/day” group, N = 57 patients). Finally, in the subgroup of patients treated in the advanced or metastatic disease setting (“first-line” group, N = 45 patients), the association between PFS and imatinib (and norimatinib) exposure was investigated.

### 2.4. Data Collection

At the end of the study period, relevant clinical data were retrieved from the patients’ medical records in collaboration with the treating oncologists. Collected data included demographic characteristics, co-morbidities, tumor-related variables (primary tumor site, metastatic sites, surgical history, c-KIT mutation status, and risk category), details of imatinib treatment, treatment-related toxicities, and tumor response. Toxicities were graded according to the Common Terminology Criteria for Adverse Events (CTCAE) version 5, while treatment response was assessed according to the Response Evaluation Criteria in Solid Tumors (RECIST). Dates of GIST diagnosis, imatinib treatment initiation and discontinuation, disease progression, death, or last follow-up were also recorded.

### 2.5. Statistical Analysis

Statistical analyses were performed using STATA software (version 14.2). Imatinib and norimatinib C_min_ were summarized for each patient as the median value of all samples collected at the same daily dose and are reported together with the interquartile range (IQR; Q1–Q3). For patients treated at multiple dose levels (e.g., 400 mg/day and 800 mg/day), a separate median C_min_ was calculated for each dose and used in the association analyses with the relevant study endpoints.

Associations between imatinib exposure and clinical–demographic characteristics were evaluated using non-parametric tests. The two-sample Wilcoxon rank-sum (Mann–Whitney) test was applied for comparisons involving two categories, whereas the Kruskal–Wallis equality-of-populations rank test was used for variables with more than two categories. To control for multiple testing, *p*-values were adjusted using the Bonferroni correction.

The relation between toxicity and C_min_ was analyzed with random-effects generalized least squares (GLS) regression to take into account within-individual repeated measures.

PFS was estimated using the Kaplan–Meier method. Patients who discontinued treatment, were lost to follow-up, or died without documented disease progression were censored at the date of last assessment. PFS analyses were stratified according to imatinib exposure (patient median C_min_ above or below: (i) the median exposure of the patients treated in a first-line setting [885 ng/mL]; (ii) the literature-reported target thresholds of 1100 ng/mL and 760 ng/mL).

To assess the association between imatinib exposure and clinically relevant toxicity the median exposure of toxic samples was compared with the median exposure of non-toxic samples. When no sample was available within the predefined time window, the patient-specific median C_min_ at the corresponding dose level was used. Differences in exposure between samples with and without clinically relevant toxicity were assessed using the Mann–Whitney test.

## 3. Results

### 3.1. Patient Characteristics

A total of 63 patients diagnosed with GIST and fulfilling all the inclusion criteria of the present study were enrolled in the aforementioned trials between February 2018 and November 2024. Patient characteristics are summarized in Table 1. The median age was 59 years at diagnosis (range, 31–81) and 64 years at study enrolment (range, 37–83), with a slight male predominance (56%). At the time of enrolment, 33 patients were already receiving imatinib therapy, whereas 30 patients were prospectively followed from treatment initiation across different clinical settings (1 neo-adjuvant, 10 adjuvant, 15 first-line, and 4 s-line). In patients followed from treatment initiation, the median time to first C_min_ assessment was 32 days (range, 13–138). For patients already on treatment at enrolment, the median time to first C_min_ measurement was 42 months (range, 9–227). The overall median follow-up duration was 19 months (range, 1–97). Most patients were treated in a first-line setting at enrolment (68%), followed by the adjuvant setting (21%). A subset of patients was followed across multiple treatment settings over time: 1 patient from neo-adjuvant to first-line therapy, 2 patients from adjuvant to advanced disease, and 10 patients from first- to second-line therapy.

### 3.2. TDM Samples

**Figure 2:**
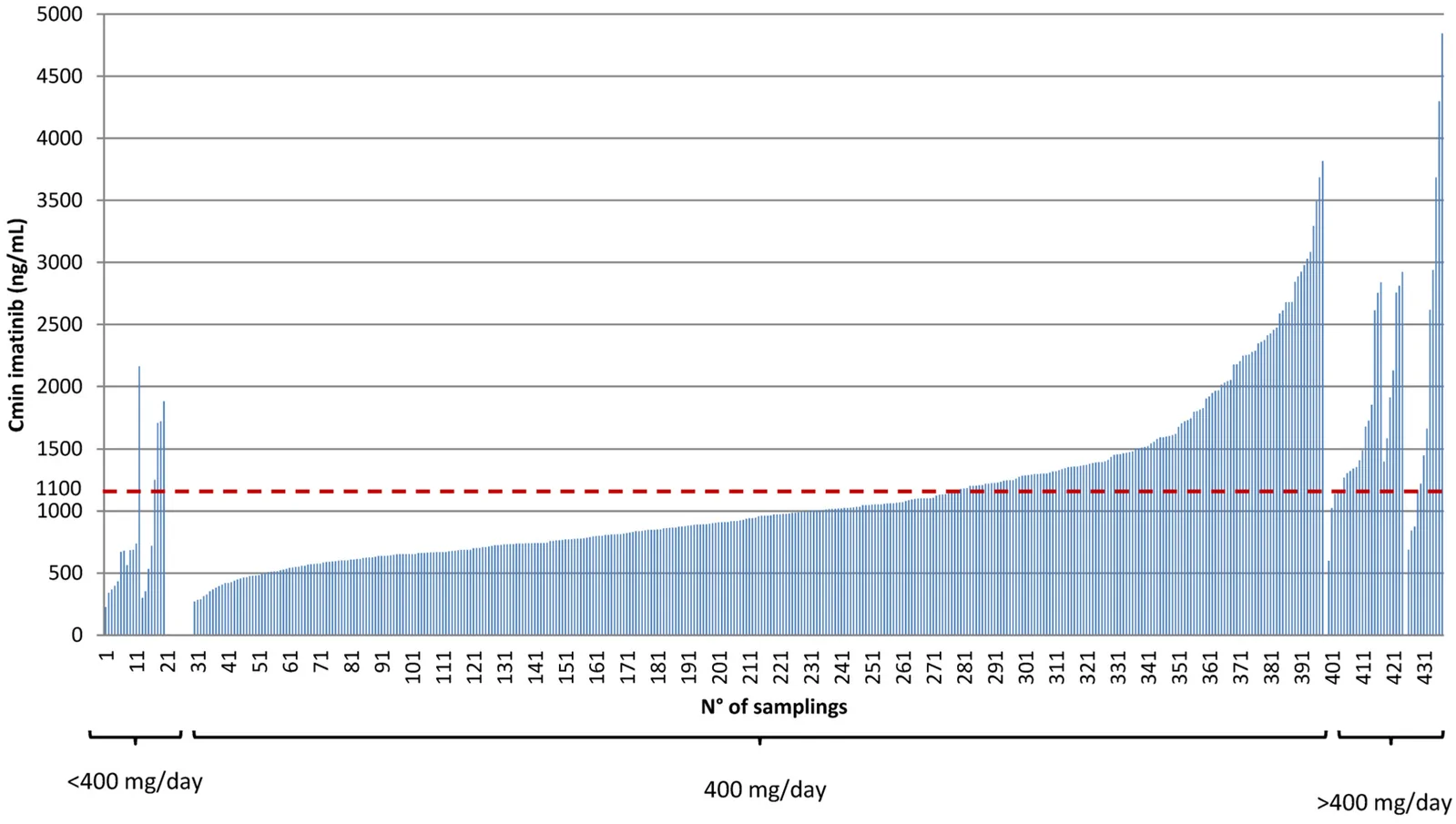
Graphic representation of imatinib C_min_ distribution among study samples divided according to imatinib doses (<400 mg/day, 400 mg/day, >400 mg/day). The dashed red line represents the C_min_ target recommended by the IATDMCT guidelines.

A total of 437 blood samples from 63 patients treated with imatinib at 100–800 mg/day were collected for TDM, with a median of 6 samples per patient (range, 1–19). The median time from last dose to blood sampling was 23 h (IQR, 19.9–24.8; range, 2.5–50.5) in patients receiving imatinib once daily and 13.5 h (IQR, 12.0–16.0; range, 2.5–30) in those receiving twice-daily dosing. Furthermore, in 344 of the 437 samples (79%), the interval between the last dose and blood sampling was within the acceptable time window recommended by Wang et al. [21] for estimating imatinib trough concentrations using the proposed algorithm (i.e., ±6 h: 18–30 h post-dose for once-daily dosing and 6–18 h post-dose for twice-daily dosing). Estimated imatinib C_min_ of each sample collected is reported in Figure 2.

Among patients treated with imatinib at doses 400 mg/day or below, the median C_min_ was 891 ng/mL (IQR, 696–1246) for imatinib and 234 ng/mL (IQR, 158–287) for norimatinib. Inter-patient variability was substantial, with coefficients of variation (CV%) of 43% for imatinib and 47% for norimatinib, while intra-patient variability was 31% and 36%, respectively. In patients treated with higher doses (>600 mg/day), the median C_min_ increased to 2613 ng/mL (IQR, 1311–2846) for imatinib and 463 ng/mL (IQR, 411–656) for norimatinib.

The median metabolic ratio (MR), defined as the ratio between norimatinib and imatinib trough concentrations, was 23.4% (IQR, 19–29%). Variability in MR was moderate, with intra-patient and inter-patient coefficients of variation of 17% and 30%, respectively. An overview of the TDM samples and exposure metrics according to dose level is reported in Table 2.

### 3.3. Exposure-Toxicity Associations

The association between imatinib exposure and clinically relevant toxicity was evaluated in the overall cohort (N = 63) by comparing the exposure levels to imatinib and norimatinib between toxic and non-toxic samples. Overall, imatinib was well tolerated, with most patients not experiencing clinically relevant toxicities across the different treatment settings. The most frequent adverse events across all settings were mild (grades G1–2) and are reported in Table 3. No relevant differences in toxicity distribution were observed between males and females.

Clinically relevant toxicity was observed in 17 patients during treatment with imatinib; however, only 14 (7 males and 7 females) had at least one sample collected for TDM according to the study criteria for toxicity assessment. Clinically relevant toxicities included G3 skin rash and/or hand–foot syndrome (4/14), G3 or persistent G2 gastrointestinal toxicity (3/14), G3 anemia (2/14 cases), subjective intolerance (2/14), G2 persistent fatigue and muscle cramps (1/14), tachycardia (1/14), fluid retention and edema (1/14), and general malaise (1/14).

Following the occurrence of clinically relevant toxicity, 4 patients (8%) required temporary interruption of first-line treatment and 2 (14%) of second-line treatment. Dose reductions were managed as follows: 2 patients (4%) reduced the dose from 400 mg/day to 200 mg/day; 1 patient (2%) from 300 mg/day to alternating doses of 200/100 mg/day; 2 patients (14%) from 800 mg/day to 600 mg/day; 1 patient (7%) from 600 mg/day to 400 mg/day; and 1 patient (7%) from 800 mg/day to 400 mg/day, followed by re-escalation to 600 mg/day after two weeks. In addition, 1 patient (7%) who showed poor tolerance to imatinib 400 mg/day in the first-line setting was started at 600 mg/day in second-line and maintained this dose until disease progression.

**Figure 3:**
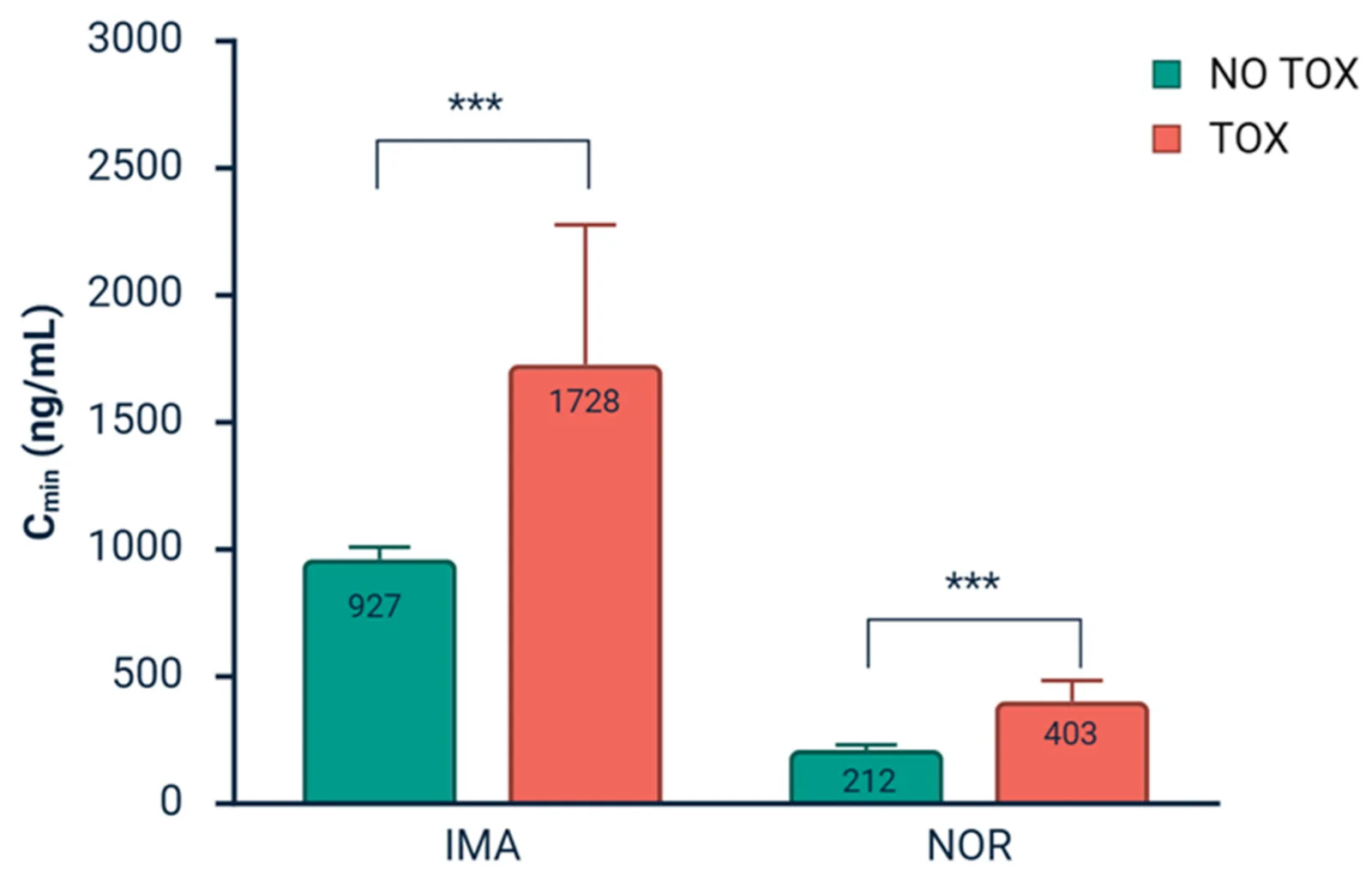
Imatinib and norimatinib exposure according to the occurrence of clinically relevant toxicity. *** indicates statistically significant differences.

Among the 437 blood samples collected across all treatment settings, 25 (6%) were classified as “toxic samples”, while the remaining 412 (94%) served as comparators (“non-toxic samples”). Toxic samples were predominantly observed in patients treated in the second-line setting, corresponding to imatinib doses ≥600 mg/day, where 8 out of 40 samples (20%) were associated with clinically relevant toxicity. In contrast, in the first-line setting, toxic samples accounted for 17 out of 288 samples (6%). No clinically relevant toxicities were recorded in the neo-adjuvant, adjuvant, or re-challenge settings during the study course. Overall, both imatinib and norimatinib exposures were significantly higher in toxic than in non-toxic samples (*p* < 0.0001). Specifically, the median imatinib trough concentration was 1728 ng/mL (IQR, 1370–2411) in toxic samples, compared with 927 ng/mL (IQR, 670–1301) in non-toxic samples. Similarly, median norimatinib concentrations were higher in toxic samples (403 ng/mL [IQR, 321–495]) than in non-toxic samples (212 ng/mL [IQR, 154–322]) (Figure 3).

When stratified by treatment setting, the exposure–toxicity association remained statistically significant in the first-line setting. Among samples collected during first-line treatment (doses ≤ 400 mg/day), toxic samples were characterized by a higher median imatinib C_min_ than non-toxic samples (1515 ng/mL [IQR, 1012–2163] vs. 892 ng/mL [IQR, 666–1249], *p* < 0.0001). Norimatinib concentrations showed a similar pattern, with higher exposure in toxic than in non-toxic samples (374 ng/mL [IQR, 291–456] vs. 207 ng/mL [IQR, 151–285], *p* < 0.0001).

In the second-line setting (doses > 600 mg/day), toxic samples exhibited a trend toward higher imatinib exposure compared with non-toxic samples (32/40, 80%), although this difference did not reach statistical significance (2232 ng/mL [IQR, 1703–2846] vs. 1469 ng/mL [IQR, 1164–2602], *p* = 0.0698). In contrast, norimatinib exposure remained significantly higher in toxic samples than in non-toxic samples (488 ng/mL [IQR, 400–758] vs. 447 ng/mL [IQR, 391–503], *p* = 0.0385).

No significant difference between exposure in toxic samples in men and women was observed (*p* = 0.452).

### 3.4. Exposure-Clinical-Demographical Characteristics Associations

In the subgroup of patients homogeneously treated with imatinib at a daily dose of 400 mg (N = 57), associations between imatinib and norimatinib exposure and clinical–demographic characteristics were explored (Table 4). The median imatinib exposure of patients in this subgroup was 891 ng/mL (IQR: 696–1246), while the median norimatinib exposure was 234 ng/mL (IQR: 158–287). In this patient group, 38/57 (67%) of patients had a median imatinib C_min_ lower than the recommended value of 1100 ng/mL.

#### 3.4.1. Stratification by Sex and Age

A significant difference in exposure was observed according to sex. Females showed higher median trough concentrations of both imatinib (1114 ng/mL, IQR 864–1318) and norimatinib (250 ng/mL, IQR 194–322) compared with males (786 ng/mL, IQR 588–1018 for imatinib; 183 ng/mL, IQR 146–263 for norimatinib), corresponding to a relative increase of +42% and +37%, respectively (*p* = 0.0018 for imatinib and *p* = 0.0249 for norimatinib). However, when patients were stratified according to body mass index (BMI), no significant differences in imatinib or norimatinib exposure were observed between normal-weight (BMI < 25 kg/m^2^) and overweight patients (BMI ≥ 25 kg/m^2^). Similarly, stratification by age alone did not reveal significant differences in exposure between patients aged <60 and ≥60 years. However, joint stratification by age and sex highlighted that in both sexes, older patients (≥60 years) tended to have higher imatinib and norimatinib exposure compared with younger patients. In particular, imatinib exposure differed significantly across age–sex subgroups (*p* = 0.0118), with younger females showing higher exposure than younger males (median C_min_ 1041 ng/mL [IQR 864–1282] vs. 668 ng/mL [IQR 588–821], *p* = 0.0068). After correction for multiple testing using the Bonferroni method (adjusted significance threshold *p* < 0.0083), this difference remained statistically significant for imatinib, while a similar trend was observed for norimatinib (*p* = 0.0881).

#### 3.4.2. Stratification by Tumor Characteristics and Co-Morbidities

With respect to tumor-related characteristics, patients with gastric GIST tended to have a non-significant lower median imatinib exposure compared with those with other primary tumor localizations (862 ng/mL [IQR 653–1116] vs. 937 ng/mL [IQR 719–1324]). Likewise, patients who had undergone surgical resection of the primary tumor showed a trend toward lower imatinib exposure compared with those who had not, while norimatinib exposure was significantly lower in surgically treated patients (*p* = 0.0172). When stratifying by surgical status, patients who had not undergone surgical treatment showed higher imatinib exposure compared with those who had received surgery (1154 ng/mL [IQR 828–2013] vs. 875 ng/mL [IQR 679–1183], *p* = 0.132), whereas no significant differences in imatinib exposure were observed between patients undergoing gastrectomy and those receiving other types of surgical resection. For norimatinib, a graded trend was observed, with progressively lower exposure across surgical categories, from no resection to gastrectomy and to other types of resection. Notably, patients with c-KIT exon 9–mutated GIST (N = 7)—who received the standard dose of 400 mg/day—showed globally higher imatinib and norimatinib exposure than those with c-KIT exon 11–mutated GIST (N = 29), (imatinib: 1370 vs. 831 ng/mL, *p* = 0.0156; norimatinib 208 vs. 322 ng/mL, *p* = 0.1801).

Finally, patients with at least one co-morbidity showed a trend toward higher exposure to both imatinib and norimatinib compared with patients without co-morbidities (imatinib: 998 ng/mL [IQR 787–1293] vs. 821 ng/mL [IQR 668–887], *p* = 0.0795; norimatinib: 244 ng/mL [IQR 181–355] vs. 165 ng/mL [IQR 146–253], *p* = 0.0529), although these differences did not reach statistical significance.

### 3.5. Exposure-Response Associations

#### 3.5.1. Efficacy Outcomes

In the subgroup of patients treated in the first-line setting (N = 45), tumor response was evaluable in 41 (91%) patients and was assessed according to RECIST criteria. The remaining 4 patients (9%) were not evaluable for response, as they had a single metastatic lesion that was surgically resected, resulting in no evidence of disease. An objective response was observed in 28 patients, including 13 complete responses (CR, 29%) and 15 partial responses (PR, 33%), yielding an objective response rate (ORR) of 62%. Stable disease (SD) was reported in 10 patients (22%), resulting in a disease control rate (DCR) of 84%. Progressive disease (PD) as best response occurred in 3 patients (7%).

The median PFS among patients who started first-line treatment during the study (N = 15) was 70 months (95% CI, 9–not reached). The median duration of response (DoR) among responders was 61 months (range, 6–73 months).

#### 3.5.2. Exposure-Efficacy Associations

The association between PFS and imatinib exposure was evaluated in 44 patients, as one patient receiving 600 mg/day was excluded from the analysis. None of the predefined imatinib exposure thresholds (i.e., 885 ng/mL, 1100 ng/mL, and 760 ng/mL) were significantly associated with PFS in our cohort (*p* = 0.5092, *p* = 0.8710, and *p* = 0.6421, respectively).

We further explored whether imatinib exposure differed according to the occurrence of disease progression (PD) within the first 18 months of therapy, corresponding to the minimum follow-up available for all evaluable patients. During this period, 4 patients—all female—experienced PD; however, one patient had c-KIT wild-type disease and was considered intrinsically insensitive to imatinib and was therefore excluded from this analysis. No statistically significant difference in imatinib exposure was observed between patients with and without progression (mean C_min_: 963 ng/mL vs. 998 ng/mL, respectively) neither in the entire first-line population (N = 44) nor in the subgroup of patients who initiated first-line therapy during the study period (N = 15, mean C_min_: 963 ng/mL vs. 1047 ng/mL in patients with and without PD, respectively).

However, stratification by exposure deciles suggested a potential gradient in progression risk. Among markedly underexposed patients (1st decile, C_min_ ≤ 500 ng/mL), 60% experienced disease progression (3/5), compared with 35% (12/34) in patients with intermediate exposure (2nd-9th deciles, C_min_ 500–1500 ng/mL), while no progression was observed in patients with C_min_ values > 1500 ng/mL (0/5) (10th decile).

## 4. Discussion

In this monocentric Italian cohort of patients with GIST, we evaluated the clinical validity of imatinib TDM and explored exposure–response relationships in terms of toxicity and efficacy, while also assessing clinical and demographic determinants of drug exposure. Our findings confirm substantial inter-individual variability in imatinib pharmacokinetics, with women achieving higher exposures than men, demonstrate a strong association between higher imatinib and norimatinib plasma levels and clinically relevant toxicity, and suggest a clinically meaningful exposure gradient in relation to disease progression. Collectively, these data highlight the clinical relevance of TDM in the personalized management of GIST.

Among patients analyzed, drug exposure was highly variable, with both inter- and intra-patient variability (43% and 31% respectively) comparable to previously published real-world cohorts (49% and 26%) [4]. Despite uniform dosing at 400 mg/day, two-thirds of patients had trough concentrations below the IATDMCT recommended target of 1100 ng/mL (median 891 ng/mL), highlighting the limitations of fixed dosing in achieving consistent systemic exposure. The proportion of underexposed patients aligns with real-world data [4,16] but contrasts with the pivotal analysis by Demetri et al. [12], in which 75% of patients had a C_min_ ≥ 1100 ng/mL and showed improved outcomes. Differences in study design, patient selection, and dose distribution—particularly the inclusion of higher-dose regimens (600 mg/day) in the clinical trial setting of Demetri et al.’s study—may partly explain this discrepancy, suggesting that exposure thresholds derived from trial populations may not fully reflect real-world pharmacokinetics.

Among the clinical-demographic variables analyzed, sex emerged as a significant determinant of exposure. Female patients exhibited significantly higher trough concentrations of both imatinib and norimatinib compared with males, in line with previous observations in GIST and CML populations [12,13,14]. Age-stratified analyses suggested a tendency toward higher exposure in older individuals, particularly women, supporting a combined effect of sex and age on drug disposition. This observation is in line with real-world data from IJzerman et al. [4], showing that patients requiring dose escalation were more often younger, underexposed, and predominantly male. These results emphasize that variability in imatinib exposure is partly driven by patient-specific factors such as sex and age, which should be considered to refine therapeutic management, as fixed dosing may not adequately account for biological differences influencing drug disposition. Incorporating sex and age into clinical decision-making, particularly in conjunction with TDM, could contribute to a more tailored treatment.

A clear exposure–toxicity relationship was observed. Toxic samples were associated with markedly higher trough concentrations (~1700 ng/mL) compared with non-toxic samples (<1000 ng/mL), supporting the existence of a relatively narrow therapeutic window, in which excessive exposure substantially increases the risk of toxicity. The exposure level associated with toxicity in our cohort is consistent with previously published evidence in which trough levels above approximately 1500–1800 ng/mL were predictive of clinically significant adverse events [18]. Similar exposure–toxicity relationships have been reported in both GIST and CML populations [13,14,15,17,19]. These data further emphasize the value of TDM in identifying patients at risk of overexposure and preventing avoidable toxicity.

In this cohort, no statistically significant association was observed between imatinib exposure and PFS across predefined concentration thresholds or when comparing patients with and without early disease progression, despite prior studies suggesting a positive exposure–response relationship [12,16]. Similar findings have been reported by IJzerman et al. [4], who also did not observe a significant association between imatinib exposure and PFS. The absence of a significant association in our analysis may reflect limited statistical power due to the small sample size and low number of progression events. In addition, the inclusion of patients who had been receiving imatinib for several years before study entry may have introduced survivorship bias, potentially enriching the cohort with patients with more favorable disease biology and treatment tolerance. Moreover, nearly 30% of patients lacked c-KIT mutational characterization, which may have further limited the interpretability of the exposure–response analysis, as different molecular subtypes are known to exhibit variable sensitivity to imatinib and distinct clinical outcomes. The relatively narrow exposure distribution, with most patients below the commonly proposed target of 1100 ng/mL, as well as real-world factors such as variable adherence and dose modifications, may have further reduced the ability to detect threshold effects. Conversely, stratification by exposure deciles showed a potential gradient in progression risk, with the highest rates among markedly underexposed patients (C_min_ ≤ 500 ng/mL) and no progression observed in those with concentrations >1500 ng/mL. However, these findings should be interpreted cautiously given the small sample size and limited number of events and should not be interpreted as evidence against the clinical utility of TDM.

Our cohort was broadly representative of real-world GIST populations [4,16]. Surgical history appeared to influence drug exposure, with lower concentrations observed in patients who had undergone gastrointestinal resection, particularly for norimatinib, consistent with previous reports suggesting altered absorption and metabolism after surgery [16,25]. Other variables previously associated with exposure, including BMI, comorbidities, and primary tumor site [16,26], showed only non-significant trends, likely reflecting limited sample size. Notably, higher exposure in patients with comorbidities is consistent with the hypothesis that inflammatory states may downregulate CYP450 activity, a concept supported by our previous observation of increased imatinib exposure during SARS-CoV-2 infection [27]. Patients harboring exon 9 c-KIT mutations, despite receiving 400 mg/day instead of the recommended 800 mg/day, exhibited significantly higher imatinib and norimatinib concentrations than exon 11–mutated patients, generally exceeding the 1100 ng/mL target threshold. Thus, in our cohort, adequate systemic exposure was achieved even at the lower dose. No clear pharmacokinetic explanation accounts for this finding; better treatment adherence may be hypothesized, although this remains speculative and requires confirmation in larger studies.

Some limitations should be acknowledged in this study, including the mixed retrospective–prospective design, relatively small sample size, heterogeneity in treatment settings and the lack of Bayesian estimation for individual pharmacokinetic parameters. Nonetheless, the study benefits from a comprehensive TDM dataset with repeated measurements per patient and careful clinical characterization, reflecting routine practice. Overall, this study extends and consolidates our previous experience—ranging from the identification of a CYP3A4-inducing DDI causing chronic underexposure [28], to inflammation-related increases in exposure [27], and the role of pharmacogenetic patient’s profile (including CYP450 and ABC transporters polymorphisms [6,7]—by providing cohort-level confirmation of the clinical relevance of imatinib TDM.

In conclusion, we confirm a marked inter-patient variability, a consistent effect of sex and age on exposure, and a significant association between higher plasma concentrations and toxicity. Although a statistically significant association with PFS was not demonstrated, possibly due to lack of power, these findings corroborate the potential clinical utility of monitoring imatinib plasma levels to identify patients at risk of avoidable toxicity and those showing extremely low exposures who may benefit from dose escalation. Our data support the integration of routine imatinib TDM into everyday clinical practice to support tailored treatment of patients with GIST; however, larger prospective studies are warranted to refine exposure thresholds and definitively clarify the exposure–efficacy relationship.

## Figures and Tables

**Table 1:** Patient characteristics.

Characteristic	N° (%) All Patients	N° (%) 400 mg/day	N° (%) First-Line
N° of patients included	63	57	45
Sex			
F	28 (44%)	26 (46%)	22 (49%)
M	35 (56%)	31 (54%)	23 (51%)
Age at diagnosis, mean, years (range)	59 (31–81)	57 (31–81)	56 (31–80)
Age at first sample, mean, years (range)	64 (37–83)	63 (37–83)	63 (37–83)
Therapy setting at enrollment			
Neo-adjuvant	2 (3%)	2 (3%)	1 (2%)
Adjuvant	13 (21%)	13 (23%)	2 (5%)
I line	43 (68%)	41 (72%)	42 (93%)
II line	4 (6%)	1 (2%)	--
Re-challenge	1 (2%)	--	--
Co-morbidities			
0	23 (36%)	21 (37%)	18 (40%)
1–2	27 (43%)	46 (46%)	19 (42%)
>2	13 (21%)	10 (17%)	8 (18%)
Type of comorbidities			
Hypertension	25 (40%)	21 (37%)	15 (33%)
Dyslipidemia	10 (16%)	9 (16%)	6 (13%)
Diabetes	6 (10%)	5 (9%)	3 (7%)
Hypothyroidism	6 (10%)	6 (11%)	1 (2%)
Other	11 (17%)	10 (18%)	7 (16%)
Performance status at first sample			
0	60 (95%)	54 (95%)	42 (93%)
1	3 (5%)	3 (5%)	3 (7%)
Primary GIST location			
Gastric	25 (40%)	24 (42%)	19 (42%)
Small Bowel	25 (40%)	22 (39%)	16 (36%)
Other	13 (20%)	11 (19%)	10 (22%)
Metastasis at diagnosis			
Yes	25 (40%)	23 (40%)	22 (49%)
No	38 (60%)	34 (60%)	23 (51%)
Metastasis site at diagnosis			
Liver	11 (42%)	10 (42%)	10 (43%)
Peritoneum	9 (34%)	9 (37%)	8 (20%)
Liver and peritoneum	4 (16%)	3 (13%)	3 (7%)
Other	2 (8%)	2 (8%)	1 (4%)
Surgery of primary tumor			
Yes	53 (84%)	50 (88%)	40 (89%)
Gastrectomy (total or partial)	23 (43%)	22 (44%)	17 (43%)
Other	30 (57%)	28 (56%)	23 (57%)
No	10 (16%)	7 (12%)	5 (11%)
Surgery of metastasis/local relapse			
Yes	18 (34%)	17 (36%)	16 (36%)
No	35 (66%)	30 (64%)	29 (64%)
Kit Mutation			
Yes	41 (65%)	38 (67%)	29 (64%)
Exon 11	32 (78%)	29 (76%)	21 (72%)
Exon 9	7 (16%)	7 (18%)	6 (21%)
Exon 10, 13 or 17	3 (6%)	3 (9%)	3 (9%)
Unknown	1 (2%)	1 (3%)	1 (3%)
No †	4 (6%)	4 (7%)	3 (7%)
Unknown	18 (29%)	15 (26%)	13 (29%)
Primary tumor dimensions			
2–5 cm	4 (9%)	4 (10%)	4 (13%)
5–10 cm	15 (32%)	13 (31%)	9 (28%)
>10 cm	27 (59%)	25 (59%)	19 (59%)
Mitotic rate			
<5/50 HPF	15 (24%)	13 (23%)	9 (20%)
>5/50 HPF	24 (38%)	23 (40%)	19 (41%)
Unknown	24 (38%)	21 (37%)	18 (39%)
Risk category			
High	40 (63%)	38 (67%)	30 (67%)
Intermediate	6 (10%)	5 (9%)	3 (7%)
Low	3 (5%)	2 (3%)	2 (4%)
Unknown	14 (22%)	12 (21%)	10 (22%)
Body Mass Index (BMI)			
<18.5	1 (2%)	1 (2%)	1 (2%)
18.5–25	20 (32%)	20 (35%)	16 (36%)
25–30	17 (27%)	17 (30%)	13 (29%)
>30	7 (11%)	6 (10%)	4 (9%)
Unknown	18 (29%)	13 (23)	11 (24%)

† one patient with wild-type c-KIT was bearing a somatic mutation in Neurofibromatosis type 1 (NF1), which can enhance the probability of developing GIST, with positive staining for Succinate Dehydrogenase (positive SDHB).

**Table 2:** TDM samples.

Variable	N°	Imatinib (ng/mL), Median (IQR Range)	Norimatinib ng/mL), Median (IQR Range)
N° of samplings	437		
N° samplings per patient, median (range)	6 (1–19)		
C_min_ at ≤400 mg/day			
Patients	57		
Samplings	399		
Mean (st. dev.)		1101 (±602)	257 (±143)
Median (IQR) on patients		891 (696–1246)	234 (158–287)
C_min_ at >600 mg/day			
Patients	11 †		
Samplings	38		
Mean (st. dev.)		2421 (±1175)	549 (±227)
Median (IQR) on patients		2613 (1311–2846)	463 (411–656)
Coefficient of Variation IMA			
Inter-patient		43.2%	47.3%
Intra-patient		30.9%	36.2%
Metabolic Ratio, MR (C_min_ NOR/C_min_ IMA)			
Mean (st.dev.)		24.6% (±7.8)	
Median (IQR)		23.4% (19.0–29.0)	
Coefficient of Variation MR			
Inter-patient		29.5%	
Intra-patient		16.6%	

† includes patients who switched from 400 to >600 mg/day.

**Table 3:** Most frequent adverse events of any grade experienced by patients during their treatment with imatinib.

Toxicity Type	Adjuvant—N° of Patients (%)	First-Line—N° of Patients (%)	Second-Line—N° of Patients (%)
Edemas (periocular, peripheral or both)	11/25 (44%)	40/51 (78%)	7/14 (50%)
Gastrointestinal disorders (nausea, vomiting, diarrhea, loss of appetite)	11/25 (44%)	39/51 (76%)	6/14 (43%)
Musculoskeletal toxicity (muscle cramps or arthralgia)	9/25 (36%)	34/51 (67%)	5/14 (36%)
Eye toxicity	5/25 (20%)	11/51(22%)	1/14 (7%)
Skin toxicity (skin rash or HFS)	3/25 (12%)	9/51 (18%)	3/14 (21%)
Asthenia	3/25 (12%)	17/51 (33%)	5/14 (36%)
Hematological toxicity	3/25 (12%)	14/51 (27%)	7/14 (50%)
Other	6/25 (24%)	24/51 (47%)	10/14 (71%)

The data presented in this table include toxicity events observed throughout the entire treatment course. Consequently, patients who experienced toxicity in multiple settings were counted multiple times.

**Table 4:** Imatinib and norimatinib exposures according to clinical-demographical characteristics.

Variable	N° (57)	Imatinib (ng/mL), Median (IQR Range)	Norimatinib ng/mL), Median (IQR Range)
C_min_ according to sex			
F	26	1114 (864–1318)	250 (194–322)
M	31	786 (588–1018), ***p*** **= 0.0018**	183 (146–263), ***p***** = 0.0249**
C_min_ according to age			
<60 years	23	887 (696–1275)	237 (146–263)
≥60 years	34	925 (679–1246), *p* = 0.8452	221 (159–369), *p* = 0.5582
C_min_ according to age and sex			
F, ≥60 years	12	1169 (885–1512)	262 (201–448)
F, <60 years	14	1041 (864–1282)	250 (183–287)
M, ≥60 years	22	813 (654–1049)	185 (156–271)
M, <60 years	9	668 (588–821), ***p *= 0.0118**	157 (140–213), *p* = 0.0881
C_min_ according to BMI (N = 44)			
<25 kg/m^2^	21	930 (737–1282)	247 (163–306)
≥25 kg/m^2^	23	891 (786–1067), *p* = 0.5970	237 (168–271), *p* = 0.4829
C_min_ according to tumor localization			
Gastric	24	862 (653–1116)	225 (153–283)
Other	33	937 (719–1324), *p* = 0.2193	234 (158–322), *p* = 0.7835
C_min_ according to surgery			
N	7	1154 (828–2013)	369 (184–495)
Y	50	875 (679–1183), *p* = 0.132	211 (156–263), ***p* = 0.0172**
C_min_ according to type of surgery			
N	7	1154 (828–2013)	369 (184–495)
Gastrectomy	22	862 (653–1183)	225 (146–279)
Other	28	892 (708–1204), *p* = 0.2231	189 (156–259), *p* = 0.056
C_min_ according to c-KIT mutations			
Exon 11	29	831 (654–1049)	208 (158–369)
Exon 9	7	1370 (1110–1536), ***p* = 0.0156**	322 (248–357), *p* = 0.1801
Other	1	679	151
Unknown	16	877 (702–1199)	foods-4489197.docx
Wild-type	4	1032 (919–1145)	246 (226–257)
C_min_ according to N° of co-morbidities			
0	21	821 (668–887)	165 (146–253)
>0	36	998 (787–1293), *p* = 0.0795	244 (181–355), *p* = 0.0529

Statistically significant *p* values are reported in bold.

## Data Availability

The data that support the findings of this study are available from the corresponding author upon reasonable request.

## Abbreviations

The following abbreviations are used in this manuscript:

ABC	ATP Binding Cassette
BMI	Body Mass Index
CML	Chronic Myeloid Leukemia
CR	Complete Response
CRO	Centro di Riferimento Oncologico
CV	Coefficient of Variation
CYP450	Cytochrome P 450
DCR	Disease Control Rate
DDI	Drug–Drug Interaction
DoR	Duration of Response
G	Grade
GIST	Gastrointestinal Stromal Tumor
GLS	Generalized Least Squares
IATDMCT	International association of Therapeutic Drug Monitoring and Clinical Toxicology
IQR	Inter-Quartile Range
LC–MS/MS	Liquid Chromatography coupled with tandem Mass Spectrometry
MR	Metabolic Ratio
ORR	Objective Response Rate
OS	Overall Survival
PD	Progressive Disease
PFS	Progression Free Survival
PR	Partial Response
RECIST	Response Evaluation Criteria in Solid Tumors
SARS-CoV-2	Severe Acute Respiratory Syndrome Coronavirus 2
SD	Stable Disease
TDM	Therapeutic Drug Monitoring
